# The Other Mechanisms by Which the *Rht* Genes Improve the Harvest Index of Wheat

**DOI:** 10.3390/plants11212837

**Published:** 2022-10-25

**Authors:** Celestin Ukozehasi, Eric S. Ober, Howard Griffiths

**Affiliations:** 1 School of Agriculture and Food Sciences, University of Rwanda, Kigali 6605, Rwanda; 2 Department of Genetics and Breeding, National Institute of Agricultural Botany, 93 Lawrence Weaver Road, Cambridge CB3 0LE, UK; 3 Department of Plant Sciences, University of Cambridge, Downing Street, Cambridge CB2 3EA, UK

**Keywords:** *Rht*, wheat, *HI*, grain number, *SLA*, MRT of N

## Abstract

Uncovering the mechanism that underlies the relationship between crop height and grain yield would potentially inform the strategies for improving wheat with optimal height. The aim of the research reported here was to identify the attributes able to produce wheat yield increases in *Rht* genotypes without further straw-shortening. Attention was given to examination in a controlled environment the question of the mechanistic foundation that determined the relationship between wheat height and yield in lines (*Rht-B1b*, *Rht-D1b*, *Rht-B1c*, *Rht-D1c*) compared to wild types in Mercia background. In addition to height reduction, this research revealed three other mechanisms by which the *Rht* genes may also improve the Harvest Index (*HI*) of wheat: (i) low Specific Leaf Area (*SLA*), (ii) increased Mean Residence Time (*MRT*) of Nitrogen (*N*), and (iii) increased grain number on spike.

## 1. Introduction

The height reduction arising from straw-shortening by reduced height (*Rht*) genes has long been proposed as the driver of the increases in harvest index (HI) of wheat [1,2,3,4,5]. These genes cause the accumulation of growth-repressing DELLA proteins, resulting in semi-dwarf plants resisting lodging. One of the main attributes behind the increased wheat yield in the past had been plant height, which has been systematically reduced as an immediate result of the introgression of *Rht* genes [6,7,8]. However, a number of comparative studies have shown that wheat yield is reduced when plant are shortened too much, thus delimiting a range of plant height to optimum yield [9,10]. Therefore, the likelihood of further increasing wheat yield through additional reductions in plant height is rather low as several studies have indicated that most modern wheat cultivars have reached the optimal heights [3,4,11,12].

The worldwide adoption of the *Rht* genes in wheat has been underway since the early 1960s [13,14]. These genes have been effective in reducing plant height by decreasing the sensitivity of vegetative tissues to endogenous gibberellic acid [15,16]. The mechanisms by which the *Rht* genes effect a reduction in plant height are relatively well understood [17]. According to Wilhelm et al. [18], the *Rht* genes encode copies of the *DELLA* protein, a growth repressor, and each *Rht* gene contains a single nucleotide polymorphism that introduces a premature stop codon. The resulting *DELLA* proteins have a reduced sensitivity to gibberellic acid (*GA*): because the *Rht* genes affect *GA*-signalling and *GAs* are involved in many development processes. The *Rht* genes have a range of effects on plants, including the reduced coleoptiles length, decreased internode length, and shorter plant height [19,20,21,22,23].

The genetics of final plant height is known to be complex, being determined by many genes. Of 21 chromosomes, 17 were found to determine genetic variation for height [24]. According to Worland [25], it is possible to classify genes for height into those which increase or promote height and those which reduce or suppress this character.

Related to their response to exogenously applied gibberellins *(GAs*), dwarf mutants can be divided into two categories [26]: (i) *GA*-sensitive mutants, where the absence or modified spectrum of endogenous gibberellins results in a dwarf plant and in which normal growth can be restored by *GA* application; and (ii) *GA*-insensitive mutants, that show a reduced response or complete insensitivity to applied *GA*. According to Gale and Gregory [27], the *GA*-insensitive mutants exhibit a reduced internodes length without reducing the length of the spike.

The *Rht-B1b, Rht-D1b, Rht-B1c,* and *Rht-D1c* are among the twenty or so major genes affecting the plant height in wheat [28,29]. These four genes are genetically related, with *Rht-B1b* and *Rht-B1c* being alleles at a locus on chromosome *4B* and *Rht-D1b* and *Rht-D1c* being alleles at a locus *4D* [18,30,31]. The greatest reductions in height are associated with *Rht-B1c* and *Rht-D1c* [19,32].

The investigation presented in this paper aimed to identify the traits by which Rht genes would produce wheat yield increases without further straw-shortening in lines (*Rht-B1b*, *Rht-D1b*, *Rht-B1c*, *and Rht-D1c*) and compared to the “Wild type” in the Mercia background.

## 2. Material and Methods

**Figure 1:**
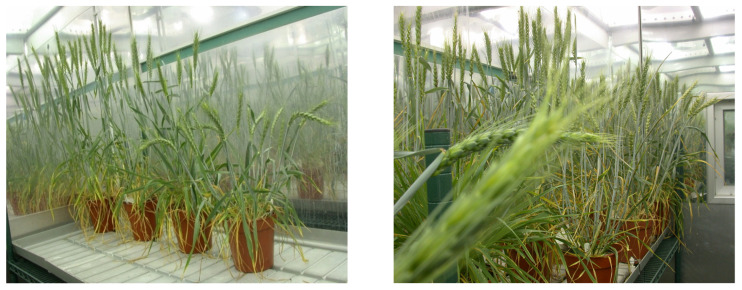
The Rht plants (*Rht-B1b, Rht-D1b, Rht-B1c, Rht-D1c*) and Mercia in pots in the growth cabinet during the experiment. Water was supplied automatically.

The measurements were collected on a four set of near isogenic lines (NILs) in the Mercia background: *Rht-B1b* (formerly *Rht1*) and *Rht-B1c* (formerly *Rht3*) being alleles on *B-Genome*; *Rht-D1b* (formerly *Rht2*) and *Rht-D1c* (formerly *Rht10*) being alleles on *D-Genome*; and wild type (Figure 1). The seeds were supplied to National Institute of Agricultural Botany (NIAB, UK) from the John Innes Centre (JIC, UK) germplasm resources unit. The vernalization requirement of winter adapted germplasm was satisfied at NIAB with 8 weeks of cold treatment in a vernalizing cabinet at 6 °C with an 8 h photoperiod, applied to 2-week-old seedlings for 6 weeks.

Thereafter, the seedlings were moved to the plant growth facility (PGF). There, the plants were placed into controlled environment growth cabinet (Conviron Ltd., Winnipeg, Canada) (Figure 1). Individual seedlings were transplanted into pots of 14 cm height, 11 cm base-width, and of 16 cm diameter at the top and filled with Levington’s *M3* compost (Scotts Professional, Suffolk UK). The pots were then arranged in randomized blocks with six replicates.

During the entire growth period, the climate in the cabinet was set at a temperature of 25 °C/23 °C (Day/night); a *PAR* of 1200 µmol photons m^−^² s^−^¹ (measured at plant height); relative air humidity 40%; 400 µmol CO_2_ s^−^¹; and a 16 h day length. Two automatic watering regimes of 3 min duration were used during the experiment. Until anthesis, the watering was achieved by thoroughly wetting capillary matting underneath the pots every 8 h, and the second regime was set at *GS65*, where watering was only supplied at moisture limit of 50% in pot and the irrigation was suspended at *GS83.* A basal fertilizer, Osmocote *14-9-11*, was added at the rate of two tablets per pot at *GS20*.

### 2.1. Plant Height Measurement

The plant height was measured at GS87 as the length (in cm) of the main tiller culm from the soil surface to the top of the spike. Similarly, the peduncle length (uppermost internode of the stem) was scored at GS87, as the length (cm) from the last node on the stem to spike collar.

### 2.2. The A/C_i_ Responses Curve Measurement and A_max_ Determination

The A/C_i_ response curve was measured on a fully expanded flag leaf of the main tiller (2 leaves per plant and 4 replicates per line) at GS65 using a LI-COR-6400XT set in auto-programme mode. The parameters were set in the Li-COR as the relative humidity to 60%, the block temperature at 25 °C, the CO_2_ reference to 400 ppm, the PAR to 1200 µmol quanta m² s^−^¹, and the ambient CO_2_ value was set as (all in ppm): 50, 100, 150, 250, 350, 500, 700, 900, 1200, 700, and 400. Once the measurement was completed, the data was downloaded from the LI-COR 6400XT to the computer. The data collected from the A/C_i_ response curve was entered into a programme called “Photosyn assistant windows software for analysis of photosynthesis, version 1.1” (Dundee Scientific, Dundee, UK) to derive A_max_.

### 2.3. Specific Leaf Area (cm² g^−^¹) Measurement

The same flag leaf samples on which gas exchange measurements was taken, were collected for specific leaf area (SLA) measurement. The leaf was cut from plant, rapidly wrapped in moist paper, placed in plastic bag, put in cool box, and taken to the laboratory for further measurements.

In the laboratory, each leaf was recut under distilled water to remove the petiole and placed immediately into a tube filled with distilled water and stored in refrigerator at 4 °C for 6 h to ensure the full rehydration of the leaves [33]. After this period, the leaf blade was taken out of the tube and blotted dry with tissue paper to remove any surface water. The leaf area (*LA*) on one side of the leaf was measured with ImageJ (version 1.42q, National Institute of Health, Bethesda, MD, USA). Then, the sample was oven dried in a paper envelope at 75 °C for 24 h. The leaf dry weight (*DW*) was obtained by reweighing the sample on micro-balance after oven drying.

The SLA was calculated as the ratio of leaf area (*LA*) to dry weight (*DW*) following Ukozehasi and Griffiths [34]:

(1) $$\mathrm{SLA}\;({\mathrm{cm}}^{2}g^{-1})=\frac{LA\;\left(cm^{2}\right)}{Dw\;\left(g\right)}$$

### 2.4. Carbon Discrimination (Δ^13^C) Measurement

At the Godwin laboratory (Cambridge University, Cambridge, UK), the dried ground leaf samples weighed (1 mg) into a tin capsule were analysed for δ^13^C using a Costech elemental analyser attached to a Thermo Delta V mass spectrometer in continuous flow mode. The mass spectrometer software measures the ^12^C/^13^C ratio. Reference standards from IAEA (International Atomic Energy Agency) in Vienna are also run at intervals throughout the sequence and these values are used to calibrate to the international standards for δ^13^C PDB.

The *δ*^13^*C* value was used to compute the Δ^13^C following Farquhar et al. [35];

(2) $$\Delta^{13}C=(\frac{\delta ¹³Ca-\delta ¹³Cp}{1+\delta ¹³Cp})/1000$$

where the *δ*^13^*C_a_* is the delta value of C in the air and the *δ*^13^*C_p_* is the delta value of C in the sample.

### 2.5. The Yield Components and Harvest Index Measurements

In grain crop-producing tillers, such as wheat, the yield components are: tillers with seeds bearing spikes, grain number, and kernel (grain) weight.

The tiller number per plant was obtained as a count of tiller with seeds bearing spikes, and the average of the number of tillers per plant for each line was computed. The whole plant biomass (including the roots) was placed into an envelope and transported to the laboratory of physiological ecology (University of Cambridge, Cambridge, UK), where the samples were freeze dried for 48 h in a Modulyo 4K Freeze dryer (Edwards High Vacuum International, West Essex, UK). Thereafter, the sample was weighed before and after threshing.

The harvest index (HI) was calculated as the ratio of grain yield to the whole plant dry weight (including roots) [36]. The grain number per spike was determined by the total number of kernels of the spikes divided by total number of spikes [4]. The grain weight was calculated as the thousand grain weight (TGW) divided by 1000 [37].

### 2.6. The ^1^⁵N Labeling and Sampling

The ^15^N label was applied to the plants in the growth cabinet. In total, 60 plants in pots were involved: 30 plants labelled with ^15^N and the other 30 plants for the control. At GS65, 50 mL of 2.5 mmol L^−^¹ ^15^N-labelled KNO_3_ (99.97%) was applied to each pot of the 30 pots (6 replicates per line), after BassiriRad and Caldwell [38]. After the labelling, the control and the labelled plants were treated equally.

For the recovery of ¹⁵N in the plant parts, half of the labelled plants were harvested 24 h after the application of ^15^N label. Similarly, half of the control plants were harvested as well. The other half of ^15^N labelled plants were retained for *MRT* of *N* measurement and harvested at *GS87*. Any unused ^15^N by the labelled plants after 24 h was removed by flushing four times with 2.5 L (10 L) of tap water in each pot. The tillers with spike in each pot were harvested and dissected into roots, stem, leaves, and spikes. All root samples were triple rinsed with deionized water.

Thereafter, a composite sample for each plant component was collected per replicate, weighed for fresh mass, and immediately frozen in liquid nitrogen. Then, the samples were freeze-dried for 48 h in Modulyo 4K Freeze dryer (Edwards High Vacuum International, West Sussex, UK). After then, they were weighed for dry mass and ground using ball mill (MM200 Mixer Mill, Glen Creston Ltd., Stanmore, UK). For each sample, a sub-sample of 1 mg was weighed into tin capsule and analysed for total *N* and ^15^N at the Godwin laboratory (University of Cambridge, Cambridge, UK).

### 2.7. The ^15^N-Enrichment Calculations

With the ^15^N data, we calculated the *MRT* of nitrogen (*N*), the *N* productivity, the nitrogen-use efficiency (*NUE*), and the nitrogen harvest index (*NHI*). The ^15^N values in plant parts (root, stem, leaf, and spike) were converted to the absolute isotope ratio (*R*) and the molar fractional abundance (*F*) following Teste et al. [39];

(3) $$R\;sample=\left[\;\left(\frac{\delta^{15}N}{1000}\right)+1\right]\times R\;standard$$

The absolute value (0.003678) of the natural abundance of ^15^N in atmospheric N_2_ was used as *R* standard.

(4) $$F=\frac{R\;sample}{R\;sample+1}$$

Then the mass-based fractional abundance (MF) was calculated as:

(5) $$MF=\frac{F\times 15}{\left[\left(F\times 15\right)+\left(1-F\right)\times 14\right]}$$

The sample *MF* values resulting from the ^15^N labelling were calculated by subtracting the *MF* of the control tissue from the *MF* of enriched sample, resulting in change in *MF* (Δ*MF*). Then, the excess sample tissue ^15^N was calculated as:

*Excess ¹⁵N* (*mg*) = *Tissue N concentration* × *tissue mass* × Δ*MF* (6)

Thereafter, we expressed the enrichment of the plant as the *Excess* ¹⁴*N* equivalent in order to highlight that *N* partitioning is shown in the common *N* form that the plant uses.

(7) $$Excess\;{}^{14}N=Excess\;{}^{15}N\times \left(\frac{14}{15}\right)$$

The nitrogen productivity (*NP*) was calculated according to Berendse and Aerts [40] as the rate of dry matter production per unit of enriched ^15^N in the plant (g dw mg^−^¹ N). Where dw is the dry weight.

The mean residence time (*MRT*) of ^15^N was computed as per proposed by Hirose [41] for both a steady and non-steady system;

(8) MRT of N (number of days)=N−ΔT/ΔN

where *N*^−^ and Δ*N* are, respectively, the plant *Excess* ¹⁴*N* amount at second harvest, the total *Excess* ¹⁴*N* amount recovery after 24 h, and Δ*T* is the number of days between the first and second harvest.

The nitrogen use efficiency (*NUE*) of ^15^*N* was calculated as the product of the nitrogen productivity of ^15^*N* and the mean residence time of ^15^*N* in the plant [40]:

(9) $$NUE\;\left(g\;dw\;mg^{-1}N\right)=NP\times MRT$$

The ^15^*N* harvest index (*^15^NHI*) was computed following Andersson and Johansson [42];

(10) $${}^{15}NHI=Excess\;¹⁴N\;amount\;in\;the\;grain/Excess\;¹⁴Namount\;in\;the\;whole\;plant$$

### 2.8. Statistical Analysis

All statistical analyses were performed using *SPSS 16.0* for Windows (SPSS Inc., Chicago, IL, USA). Firstly, the data was explored for the parametric assumptions of the normal distribution and homogeneity of variance using the Kolmogorov–Smirnov (*K-S*) and Levene’s tests, respectively. Then, the graphing of means was performed, using bar charts and the analysis for linear relationship was conducted by the means of scatter plots. Thereafter, the data was subjected to the partial Pearson correlation analysis. The one-way-independent *ANOVA* was performed at *p* < 0.01*,* followed by the post hoc test using Bonferroni test at significance level *p <* 0.01.

## 3. Results

### 3.1. Plant Height

**Figure 2:**
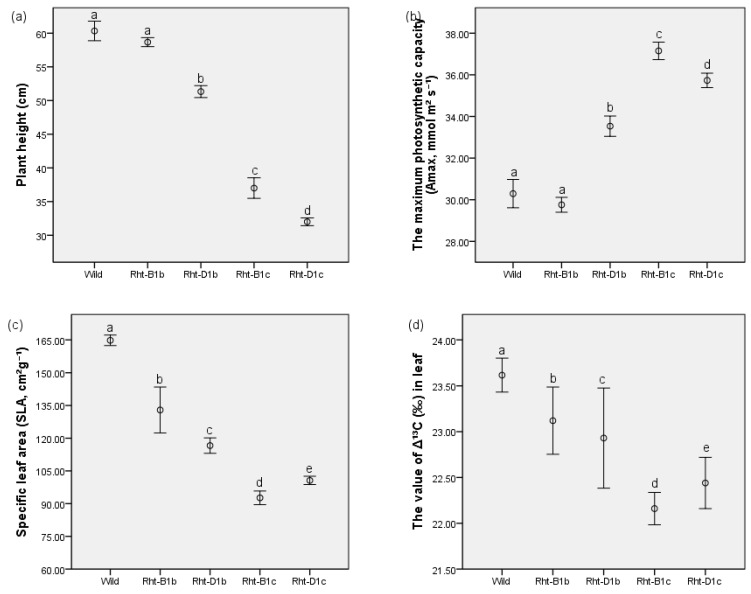
Variation of the traits of photosynthesis among the lines (Wild type, Rht-B1b, Rht-D1b, Rht-B1c, and Rht-D1c) in relation to their height: (**a**) plant height, (**b**) the maximum photosynthetic capacity, (**c**) the specific leaf area, and (**d**) the value of ∆¹³C in leaf. Values are means ± SE (n = 8). The bars showing the same letters indicate that their mean values do not statistically differ significantly (*p <* 0.01).

The plant height varied cultivars—the tall wild type at 60.3 ± 1.5 cm and the shortest *Rht-D1c* at 32 ± 0.6 cm tall. The *Rht-B1c* was 37 ± 1.5 cm, *Rht-B1b* was 58.7 ± 0.7 cm, and the *Rht-D1b* was 51.3 ± 0.9 cm. The height of *Rht-B1b* and the wild type did not differ significantly at *p <* 0.01 (Figure 2a).

### 3.2. The Effects of Straw-Shortening on Photosynthesis

The presence of *Rht* genes was associated with an increase in the maximum photosynthetic capacity (Amax) (33.5 ± 0.5 µmol; 37.2 ± 0.4 µmol; 35.7 ± 0.3 µmol, all per m² s^−^¹; for *Rht-D1b*; *Rht-B1c*; and *Rht-D1c*, respectively, compared to the control (30.3 ± 0.7 µmol m² s^−^¹) (Figure 2b). The *Δ¹³C* values in leaf organic matter (22.16 ± 0.18‰ and 22.44 ± 0.28‰ for *Rht-B1c* and *Rht-D1c*, respectively) and the *SLA* (92.7 ± 3.1 cm² g^−^¹ and 100.6 ± 1.9 cm² g^−^¹ for *Rht-B1c* and *Rht-D1c*, respectively) were significantly (*p* < *0*.01) lower in shorter lines (*Rht-B1c*, *Rht-D1c*) than in taller ones (23.12 ± 0.37‰ and 132.9 ± 10.5 cm² g^−^¹ for *Rht-B1b* and 22.93 ± 0.55‰; 116.6 ± 3.5 cm² g^−^¹ for *Rht-D1b*) and the wild type (23.62 ± 0.18‰; 164.8 ± 2.4 cm² g^−^¹; for Δ¹³C and *SLA*, respectively) (Figure 2c,d). The data also shows a significant decline in the maximum photosynthetic capacity (Amax) in Rht-D1c compared to Rht-B1c (Figure 2b), indicating there may be a threshold of dwarfing beyond which the Amax starts decreasing—this observation of the effect of too much straw shortening on Amax is confirmed here in the data both of SLA and *Δ¹³C* in leaf matter (Figure 2c,d).

### 3.3. The Effects of Straw Shortening on the MRT of N, Harvest Index, and Grain Number on Spike

**Figure 3:**
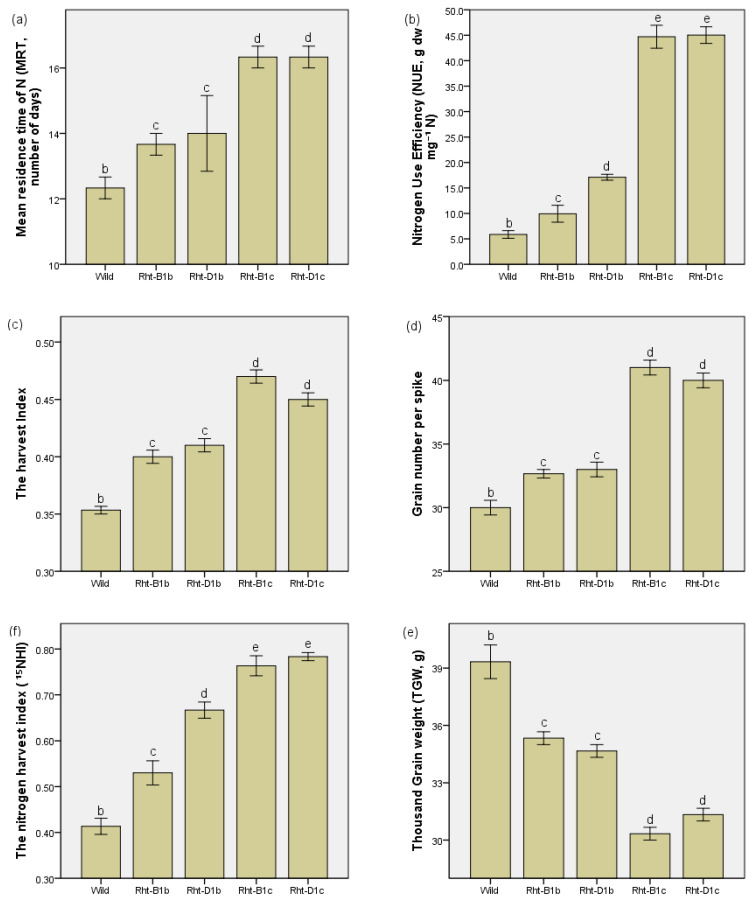
The effect of straw shortening on MRT of N, harvest index, and grain number on spike: (**a**) variation of MRT of N (number of days) by lines with different level of dwarfing, (**b**) variation of NUE of N (g dw mg^−^¹) by lines with different level of dwarfing, (**c**) the harvest index, (**d**) grain number per spike, (**e**) thousand grain weight, and (**f**) the N harvest index. Values are means ± SE (n = 6). The bars showing the same letters indicate that their mean values do not statistically differ significantly (*p <* 0.01).

The MRT of N, NUE, *HI*, grain number, and the partitioning of N to grain increased with the level of dwarfing (Figure 3a–d,f), but the straw-shortening did not influence the level of tillering (Table 1). It worth noting a slight decline in both the harvest index and grain number on spike in Rht-D1c compared Rht-B1c (Figure 3c,d), indicating again that too much straw shortening may result in reduced HI and yield. The data also show that straw-shortening may lead to a trade-off between the increased grain number on the spike and grain weight (Figure 3d,e).

The straw-shortening may lengthen the duration to anthesis (Table 1). The straw-shortening by *Rht-D1c* mostly lengthened the duration to anthesis compared to all other lines (Table 1).

The Pearson correlation analysis showed that the plant height was significantly related to the specific leaf area, maximum photosynthetic capacity, mean residence time of N, nitrogen use efficiency, harvest index, grain number on spike, grain mass, partitioning of N to grain, biomass, and days to anthesis (Table 2).

## 4. Discussion

The aim of this research was to identify the traits able to produce wheat yield increases without further straw-shortening. In addition to height reduction, this research has revealed three other mechanisms by which the *Rht* genes may also improve the harvest index (*HI*) of wheat, defined as the ratio of grain yield to total biomass: (i) low *SLA*, (ii) increased *MRT* of *N*, and (iii) increased grain number on spike.

The result of this research clearly indicated that the *Rht* genes may decrease the *SLA* and that there may be a threshold level of dwarfing beyond which further straw-shortening may result in reduced grain yield.

We found an intimate association between the level of dwarfing, the photosynthetic capacity (*A_max_*), and *SLA*. The lines with low *SLA* consistently exhibited a higher rate of *A_max_* compared to that of higher *SLA*. Combining these observed relationships enabled us to uncover the mechanistic that links straw-shortening to photosynthetic rate. A single regression accurately showed that *A_max_* is related to *SLA*, increasing with decreases in *SLA*. A similar analysis illustrated the effect of straw-shortening on both *SLA* and flag leaf *Δ¹³C*. These results lead to the suggestion that the straw-shortening effects *A_max_* by exerting a controlling influence over *SLA*. The *Rht* genes might affect the size of *SLA*, as a result, *A_max_* would be affected. This finding corroborates with both Lecain et al. [43] and Keyes et al. [15] who observed that the flag leaf of dwarf wheat had a thicker leaf than the tall type. In agreement with this finding, Morgan et al. [44] found more chlorophyll, protein, and Rubisco content per unit leaf area in the dwarf than the tall isolines and ascribed the effect of dwarfing genes on photosynthesis to a greater density of cells capable of photosynthesis. We therefore propose that straw-shortening may improve *A_max_* through the effects of lowering the *SLA.*

Increasing the *MRT* of *N* is likely the other mechanism by which straw-shortening improves the *HI* of wheat: the results of this research have consistently shown that the shortest lines (Rht*-B1c* and *Rht-D1c*) exhibited an increased *MRT* of *N* than the relatively tall lines. Selection for increased *MRT* of *N* may also be important in enhancing the stay-green of the leaf and could therefore provide an additional route to increasing photosynthetic efficiency. The increased *MRT* of *N* also is a characteristic possibly related to *NUE*, as indicated by the results of this research. The high *MRT* of *N* has been proposed to be an important indicator of the plant regarding N stress [45]. Therefore, the results of this research lead us to suggest that the selection for high *MRT* of *N* may be one of the strategies necessary to meet the challenge of low nitrogen fertilizer input cost.

Based on results of this study, it is likely that the *Rht* genes also benefit the *HI* via the effect of grain number on spike. The increases in grain number on spike were consistently associated with the level of dwarfing with *Rht-B1c* and *Rht-D1c*, exhibiting the highest grain number compared to the other lines. Some of the possible explanations of the increased grain number in *Rht* lines was proposed by Youssefian et al. [1,2], who argued that it derives from the increased partitioning of assimilates to the developing ear as a consequence of reduced demand for stem elongation, therefore resulting in improved floret fertility.

However, there may be competition effects on kernel weight associated with increases in the grain number on spike. For example, through a close examination of our results, we noted that the number of grains on the spike was partially offset by a reduction in kernel weight, indicating a trade-off between grain number and kernel weight on spike. Whether the reduced grain size is a competitive response to the increase in the number of kernels on the spike or the primary effect of straw-shortening was not clear, but we would suggest the former might be the cause. This assertion may explain the notion of reduced grain yield associated with excessive dwarfing: it is possible that extreme straw-shortening increases grain number by much far at the expense of kernel weight (resulting in lighter grains that may be caused by the greater competition for assimilates due to greater numbers on the spike). Therefore, realizing the yield advantage of *Rht* dwarfs depends upon achieving a balance between the increases in grain number and kernel weight. For this to be realized, selection for grain length in addition to grain number would be one of the strategies; evidence in support of this comes from Gegas et al. [46], who show that grain length and grain volume are controlled independently.

One of the main attributes modified to increase the *HI* was plant height, which was systematically reduced [47]. Our results indicated the increased adding effect of *Rht-B1b*, *Rht-D1b*, and *Rht-B1c* on *A_max_*, *HI*, and the grain number on spike but with relative reduction for *Rht-D1c* compared to *Rht-B1c*, thus showing there might penalty for reducing height beyond *Rht-B1c*.

The concept of *HI* was extended to the partitioning of *N*, in particular, the nitrogen harvest index (NHI), as the ratio of nitrogen in grain to total nitrogen content of the plant biomass, including roots [48]. The grain *NHI* is important trait in relation to the baking quality and nutritional value of wheat. We therefore examined the extent the straw-shortening influences the *NHI*, using the ^15^N labelling as the experimental technique. The study indicated that straw-shortening increased the *^15^NHI* linearly with the level of dwarfing.

The current research also evaluated the effects of straw-shortening on earliness of flowering. The data indicated the *Rht* genes may lengthen the duration to flowering. We speculated that the effect of straw-shortening on lengthening the time to anthesis would be the consequence of *Rht* genes lengthening the stem elongation phase. This is in agreement with Reynolds et al. [36] who suggested that the variation in the lengthening of the stem elongation phase means the cultivars may differ in their earliness of flowering.

In conclusion, the study indicated that the partitioning of *N* to spike is the characteristic related to plant height. Moreover, the selection of wheat cultivars for increased *HI* should shift focus from reduced plant height to include reduced *SLA* (for the environment with enough light); increased MRT of N, grain number, grain length, kernel weight, and partitioning of N to grain; reduced days to anthesis; and increased number of tillers.

Further research is needed to understand the mechanism by which extreme straw-shortening reduces yield. This could be supported by research on the identification of *QTL* to reduce height without reducing grain yield or seeking independent control of kernel weight. Further work should also be carried out to better understand the genetic control of *SLA* and investigate whether reliable genetic markers can be identified.

## Figures and Tables

**Table 1:** Variation in number of tillers and days to anthesis by line (Wild type, Rht-B1b, Rht-D1b, Rht-B1c, and Rht-D1c).

Line	Parameter	
	Number of Tillers (Mean ± SE, N = 60)	Number of Days to Anthesis (Mean ± SE, N = 60)
Wild type	33.3 ± 1.9	52.0 ± 0.7
Rht-B1b	24.7 ± 5.5	54.0 ± 0.6
Rht-D1b	19.7 ± 1.8	55.7 ± 0.3
Rht-B1c	21.0 ± 2.0	56.3 ± 0.2
Rht-D1c	27.3 ± 3.2	59.3 ± 0.7

**Table 2:** The Pearson correlations coefficients between measured traits across all lines. Abbreviations: *SLA*, specific leaf area; *A_max_*, maximum rate of photosynthesis; Δ^13^C, carbon isotope discrimination ratio; *# tiller*, number of tillers per plant; *# grain*, number of grains per spike; *TGW*, thousand grain weight; *DANT*, Number of days to anthesis; *Ped L.*, length of peduncle; *HI*, harvest index; *NP*, ni-trogen productivity; *NUE*, nitrogen use efficiency; *MRT*, mean residence time of N; *NHI*, nitrogen harvest index.

Parameter	*SLA*	*A_max_*	Δ¹³C	*# tiller*	*# grain*	*TGW*	*DANT*	*Ped L.*	*HI*	Biomass	*NP*	*NUE*	*MRT*	*NHI*	*Height*
*A_max_*	−0.85 ^**^														
Δ¹³C	0.80 ^**^	−0.71 ^**^													
*# tiller*	0.36	−0.34	0.23												
*# grain*	−0.85 ^**^	0.86 ^**^	−0.63 ^**^	−0.27											
*TGW*	0.94 ^**^	−0.83 ^**^	0.73 ^**^	0.36	−0.92 ^**^										
*DANT*	−0.80 ^**^	0.70 ^**^	−0.51 ^*^	−0.29	0.79 ^**^	−0.81 ^**^									
*Ped L.*	0.63 ^**^	−0.69 ^**^	0.45 ^*^	0.06	−0.90 ^**^	0.79 ^**^	−0.73 ^**^								
HI	−0.94 ^**^	0.88 ^**^	−0.69 ^**^	−0.36	0.92 ^**^	−0.96 ^**^	0.82 ^**^	−0.76 ^**^							
*biomass*	−0.92 ^**^	0.91 ^**^	−0.67 ^**^	−0.37	0.90 ^**^	−0.91 ^**^	0.89 ^**^	−0.74 ^**^	0.91 ^**^						
*NP*	−0.88 ^**^	0.92 ^**^	−0.66 ^**^	−0.24	0.95 ^**^	−0.90 ^**^	0.83 ^**^	−0.85 ^**^	0.91 ^**^	0.96 ^**^					
*NUE*	−0.86 ^**^	0.91 ^**^	−0.70 ^**^	−0.21	0.97 ^**^	−0.90 ^**^	0.79 ^**^	−0.88 ^**^	0.90 ^**^	0.93 ^**^	0.98 ^**^				
*MRT*	−0.79 ^**^	0.81 ^**^	−0.63 ^**^	−0.21	0.85 ^**^	−0.83 ^**^	0.74 ^**^	−0.84 ^**^	0.85 ^**^	0.82 ^**^	0.91 ^**^	0.89 ^**^			
*NHI*	−0.91 ^**^	0.87 ^**^	−0.62 ^**^	−0.42	0.88 ^**^	−0.92 ^**^	0.88 ^**^	−0.74 ^**^	0.93 ^**^	0.98 ^**^	0.94 ^**^	0.90 ^**^	0.84 ^**^		
*Height*	0.84 ^**^	−0.90 ^**^	0.64 ^**^	0.17	−0.94 ^**^	0.88 ^**^	−0.85 ^**^	0.87 ^**^	−0.88 ^**^	−0.95 ^**^	−0.98 ^**^	−0.98 ^**^	−0.86 ^**^	−0.90 ^**^	

^**^ Correlation is significant at the 0.01 level (1-tailed). ^*^ Correlation is significant at the 0.05 level (1-tailed).

## Data Availability

Not applicable.
